# Benefits of Sanitation

**Published:** 1881-10

**Authors:** 


### BENEFITS OF SANITATION.

London is not only the largest and the healthiest of the large cities of the world, but it is probably the only city that possesses trustworthy mortality statistics extending over a period of forty years. . . . We now know that the true London death-rate during last year did not exceed 21.5 per thousand, a lower death-rate than which has been recorded in only three of the past forty years. . .

The health of London, which was practically stationary during the thirty years ending 1870, exhibited a marked improvement during the following ten years, and it may be estimated that at least seventy thousand persons within Registration London were living at the end of the ten years who would have died had the mean death-rate of the preceding thirty years been maintained. It is satisfactory to note, moreover, that if the last decade be divided into two quinqueniads, the death-rate in the latter is considerably lower than that in the earlier five years. These facts are referred to by the registrar-general, as affording strong evidence that the sanitary efforts of recent years have not been unfruitful, more especially as the increasing density of the population in London would inevitably have led to increased mortality, if not counteracted by improved health organization.

The reference of the lower death rate of the last decennium to improved sanitation in the metrepolis, appears to be more fully justified when it is borne in mind that the saving of life is almost entirely due to diminished mortality from zymotic diseases. It is shown that this mortality, which had been all but stationary during the three preceding decennia, fell during the last decade no less than twenty-five per cent. below its previous average. . .

We are reminded that nearly nineteen thousand more persons would have died from fever during the ten years in London if the rate from this cause had remained at its previous level ; and further, that more than one hundred thousand Londoners were preserved from attacks of fever by the improved health-condition of the metropolis. The fatality of scarlet fever during the past ten years was also considerably below that recorded in any of the three preceding decades. The death-rates from measles, hooping-cough and diarrhea were also somewhat lower during the last decade, although the decline was less marked.

